# Paraoxonase-1 is related to inflammation, fibrosis and PPAR delta in experimental liver disease

**DOI:** 10.1186/1471-230X-9-3

**Published:** 2009-01-14

**Authors:** Judit Marsillach, Jordi Camps, Natàlia Ferré, Raul Beltran, Anna Rull, Bharti Mackness, Michael Mackness, Jorge Joven

**Affiliations:** 1 Centre de Recerca Biomèdica, Hospital Universitari de Sant Joan, Institut d'Investigacions Sanitàries Pere Virgili, Universitat Rovira i Virgili, C. Sant Joan s/n, 43201 Reus, Spain

## Abstract

**Background:**

Paraoxonase-1 (PON1) is an antioxidant enzyme synthesized by the liver. It protects against liver impairment and attenuates the production of the pro-inflammatory monocyte chemoattractant protein-1 (MCP-1). We investigated the relationships between hepatic PON1 and MCP-1 expression in rats with liver disease and explored the possible molecular mechanisms involved.

**Methods:**

CCl_4 _was administered for up to 12 weeks to induce liver damage. Serum and hepatic levels of PON1 and MCP-1, their gene and protein expression, nuclear transcription factors, and histological and biochemical markers of liver impairment were measured.

**Results:**

High levels of PON1 and MCP-1 expression were observed at 12^th ^week in the hepatocytes surrounding the fibrous septa and inflammatory areas. CCl_4_-administered rats had an increased hepatic PON1 concentration that was related to decreased gene transcription and inhibited protein degradation. Decreased PON1 gene transcription was associated with PPARδ expression. These changes were accompanied by increased hepatic MCP-1 concentration and gene expression. There were significant direct relationships between hepatic PON1 and MCP-1 concentrations (P = 0.005) and between PON1 and the amount of activated stellate cells (P = 0.001).

**Conclusion:**

Our results from this experimental model suggest a hepato-protective role for PON1 against inflammation, fibrosis and liver disease mediated by MCP-1.

## Background

Chronic liver diseases are characterised by the concomitant presence of oxidative stress and a severe inflammatory response [1]. The ubiquitous presence of antioxidant enzymes may represent an important defence mechanism in diminishing the burden of the pro-oxidant stimuli. Paraoxonase-1 (PON1), an enzyme with lactonase and esterase activities, is synthesized, in humans, mainly by the liver [2,3]. It hydrolyses lipid peroxides, and circulates in plasma bound to high-density lipoproteins (HDL) [4]. We have reported previously that serum PON1 activity is decreased in patients with liver diseases, while serum PON1 concentration and hepatic PON1 protein expression are increased [5-7]. We also proposed that PON1 may play a role in the regulation of hepatic parenchymal cell apoptosis [6]. More recent evidence indicates that PON1 over-expression provides strong protection against the development of experimental liver disease [8]. Conversely, low PON1 levels are associated with an enhanced sensitivity to the development of liver damage [9].

The cells responsible for the inflammatory response may vary but, usually, resident or recruited monocytes/macrophages play a key role [10]. Monocyte chemoattractant protein-1 (MCP-1) regulates the recruitment of monocytes into tissues and their subsequent differentiation to macrophages. Its expression is increased in patients with chronic inflammatory diseases, including liver impairment [11-14]. In liver cirrhosis, MCP-1 expression is up-regulated in portal tracts, epithelial cells of regenerating bile ducts, activated stellate cells and Kupffer cells [10]. This suggests that the protein may be involved in sustaining hepatic injury and fibrosis and, as such, to down-regulate the action of MCP-1 may represent a potentially effective therapeutic option.

Despite evident clinical interest, the relationships between PON1 expression and MCP-1 production in chronic liver diseases have not been studied to-date. The present study was designed to investigate the chronological sequence and quantitative relationships between PON1 expression and activity, free radical production, MCP-1 expression, and fibrosis. The model used was experimental rats with chronic liver impairment induced by CCl_4 _administration and, in which, free radical production and inflammatory cell recruitment to the liver have been extensively documented [15-18]. Also, we examined the possible rebound of genetic and pathological changes following the cessation of the hepato-toxic injury, and we explored the molecular mechanisms that may be implicated in the observed changes.

## Methods

### Experimental design

The handling of animals and the procedures described were approved by the Ethics Committee of the Rovira i Virgili University. Liver fibrosis was induced in male Wistar rats (n = 30) weighing 207 ± 9 g (Panlab, Barcelona, Spain) by twice a week intra-peritoneal (i.p.) injections of 0.5 mL of CCl_4 _diluted 1:1 (v/v) in olive oil [19]. CCl_4 _administration was continued for up to 12 weeks in a group of 18 rats; 3 subgroups of 6 animals each being sacrificed at 6, 8 and 12 weeks of CCl_4 _administration. Another group of 12 rats received CCl_4 _for 6 weeks, the toxicity-inducing agent was stopped, and 2 subgroups of 6 animals each were killed at weeks 7 and 8 (1 and 2 weeks of recovery). An additional group of 6 rats receiving only olive oil was used as a control group. All the animals were fed *ad libitum *with standard rat chow (Harlan Interfauna, Barcelona, Spain). Before sacrifice, the livers were removed under anaesthesia. Portions were fixed in 4% formaldehyde and embedded in paraffin for histology examination. The remaining portion of the liver was frozen in liquid nitrogen and stored at -80°C for subsequent biochemical and RNA expression analyses.

### Biochemical measurements

Blood was obtained by cardiac puncture and serum and EDTA-plasma samples were stored at -80°C until required for analysis. Liver tissue (30 mg) was homogenised in 500 μl of 25 mM Tris-HCl buffer (pH = 7.4) with 100 mM NaCl, and 1% Nonidet-40, in a Precellys 24 homogeniser (Bertin Technologies, Montigny-le-Bretonneux, France). Homogenisation was 1 cycle of 23 s at 6500 rpm. The esterase activity of PON1 was measured as the rate of hydrolysis of paraoxon at 410 nm and 37°C [5]. The lactonase activity was measured as the hydrolysis of 5-thiobutyl butyrolactone (TBBL) [20,21]. PON1 concentration was determined by an in-house ELISA method using a polyclonal antibody raised against a peptide derived from the sequence of mature PON1 [22]. MCP-1 concentration was analysed with a murine JE/MCP-1 ELISA development kit from Peprotech (Rocky Hill, NJ, USA). Serum aspartate aminotransferase (AST) activity, and cholesterol and triglyceride concentrations were measured with reagents purchased from Beckman-Coulter (Fullerton, CA, USA).

### Quantitative real-time PCR

A 30 mg portion of liver was lysed with 650 μL of 1× Nucleic Acid Purification Lysis Solution^® ^(Applied Biosystems, Darmstadt, Germany). RNA extraction was performed in an ABI Prism™ 6100 nucleic acid prep-station (Applied Biosystems). Reverse transcription to cDNA of 1 μg of total RNA was with random hexamer primers from Invitrogen (Carlsbad, CA, USA). Real-time PCR analysis was performed with TaqMan^® ^Low Density Arrays (Applied Biosystems) [23] using primers and probes (TaqMan^® ^Gene Expression Assays) for rat *PON1*, *MCP-1*, peroxisome proliferator-activated receptor α *(PPARα)*, *PPARδ*, and *PPARγ *genes. Thermal cycling and fluorescence detection was performed in an Applied Biosystems ABI Prism 7900 HT Sequence Detection System. Amplification involved 40 cycles with the following parameters: 2 min at 50°C, 10 min at 94.5°C and, for each cycle, 30 s at 97°C for denaturation and 1 min at 59.7°C for transcription. Analysis of gene expression values were performed using the 2^-ΔΔCT ^method. β-glucoronidase (*Gusb*) gene expression was used for normalisation. This gene has been shown not to undergo any significant change during prolonged administration of CCl_4 _[24].

### Measurement of AP-1 and Sp-1 binding activation

DNA-binding activity of Sp-1 and that of several members of the AP-1 family of nuclear transcription factors (c-Fos, FosB, c-Jun, JunB, JunD, Fra-1, and Fra-2) were analysed in liver homogenates using DNA-binding ELISA methods (Active Motif, Carlsbad, CA, USA).

### Histological and immunochemical methods

Livers were fixed in 10% phosphate-buffered formalin for 24 h at room temperature, washed twice with water, stored in 70% ethanol at 4°C, and embedded in paraffin. PON1 protein expression was assessed by immunohistochemistry using the previously described polyclonal anti-PON1 antibody [22]. MCP-1 and PPARδ protein expressions were assessed with polyclonal antibodies from Santa Cruz Biotechnology Inc. (Santa Cruz, CA, USA). Hepatic 4-hydroxy-2-nonenal-protein adducts were measured as an index of lipid peroxidation using a monoclonal antibody from the Japan Institute for the Control of Aging (Shizuoka, Japan). Macrophage staining was performed with a monoclonal antibody against the F4/80 antigen (Serotec, Oxford, UK). The amount of activated stellate cells in the liver was estimated by α-smooth muscle actin (α-SMA) immunohistochemistry [25] using an anti-SMA antibody from Novocastra (Menarini, Florence, Italy). Biotinylated secondary antibodies used in all the immunochemical methods were obtained from Vector Laboratories (Burlingame, CA, USA). Sections were counterstained with haematoxylin. Collagen content of biopsies was estimated by image analysis of the Masson's trichrome stain using an image software system (AnalySIS™, Soft Imaging System, Munster, Germany).

### Evaluation of hepatic proteolytic activity

To evaluate the hepatic proteolytic ability, we measured the cathepsin B activity in liver homogenates obtained as described above. Cathepsin B is a lysosomal cysteine protease and its levels are a reliable index of the overall hepatic proteolysis [26]. We used the Innozyme™ Cathepsin B Activity Assay Kit (Calbiochem, Gibbstown, NJ, USA). We investigated the appearance in the homogenates of the inactive procathpsin B by Western Blot using a rabbit polyclonal primary antibody against rat cathepsin B (Abcam, London, UK) which also recognises the proenzyme. Secondary antibodies used were goat polyclonal anti-rabbit immunoglobulins/HRP (Dako, Glostrup, Denmark). We also investigated the presence of anti-PON1 immunoreactive fragments in liver homogenates by Western Blot using the polyclonal anti-PON1 antibody described before [22].

### Statistical analyses

The effects of CCl_4 _administration and cessation (recovery) on the biochemical variables over multiple time periods were analysed by ANOVA. The Mann-Whitney *U *test was used to compare differences between any 2 individual time periods. The Spearman regression test was used to evaluate the degree of association between any 2 variables. Results are shown as means ± SEM. Statistical analyses were performed with the SPSS 14.0 statistical package (SPSS Inc., Chicago, IL. USA).

## Results

### Changes in variables associated with liver injury

The development of chronic liver impairment was associated with significant increases in serum AST activity, the percentage of collagen, and the amount of activated stellate cells in liver tissue. Cessation of CCl_4 _administration was associated with a partial recovery of normal liver histology and function, as evidenced by a decrease in serum AST activity and the percentage of collagen and activated stellate cells staining in the liver tissue (Table 1). Serum cholesterol and triglyceride concentrations decreased significantly with CCl_4 _administration and, in the case of cholesterol, became normalised by the end of the study. Cessation of CCl_4 _administration was associated with a partial recovery of both parameters.

**Table 1:** Effect of CCl_4 _on the liver injury

	Control group No CCl_4_	CCl_4 _administration groups			Recovery groups CCl_4 _withdrawal	
		
		6 weeks	8 weeks	12 weeks	1 week	2 weeks
Collagen (%)	0.22 (0.04)	0.97 (0.31)^a^	0.47 (0.06)^a^	3.6 (0.3)^a, e, h^	0.67 (0.09)^a^	0.73 (0.16)^a^
α-smooth muscle actin staining (%)	0.109 (0.02)	1.2 (0.3)^b^	0.41 (0.07)^b^	1.8 (0.5)^b, g^	0.21 (0.03)^a, d^	0.11 (0.02)^d, i^
Aspartate aminotransferase (μkat/L)	2.0 (0.2)	5.9 (1.2)^a^	5.4 (1.0)^a^	25.0 (7.7)^a, c, f^	2.3 (0.4)^d^	2.0 (0.3)^d^
Cholesterol (mmol/L)	0.94 (0.07)	0.70 (0.09)^a^	0.78 (0.15)	1.08 (0.14)^c^	1.00 (0.15)	1.12 (0.95)
Triglycerides (mmol/L)	2.65 (0.41)	0.63 (0.08)^b^	0.56 (0.06)^b^	0.72 (0.18)^b^	1.08 (0.13)^b^	0.89 (0.13)^b^

^a^: *p *≤ 0.05, ^b^: *p *< 0.01, with respect to the control group. ^c^: *p *≤ 0.05, ^d^: *p *< 0.01, ^e^: *p *< 0.001, with respect to 6 weeks of CCl_4 _administration. ^f^: *p *≤ 0.05, ^g^: *p *< 0.01, ^h^: *p *< 0.001, with respect to 8 weeks of CCl_4 _administration. ^i^: *p *≤ 0.05, with respect to 1 week of CCl_4 _withdrawal/recovery

### Hepatic PON1 expression is associated with MCP-1 and fibrosis

**Figure 1:**
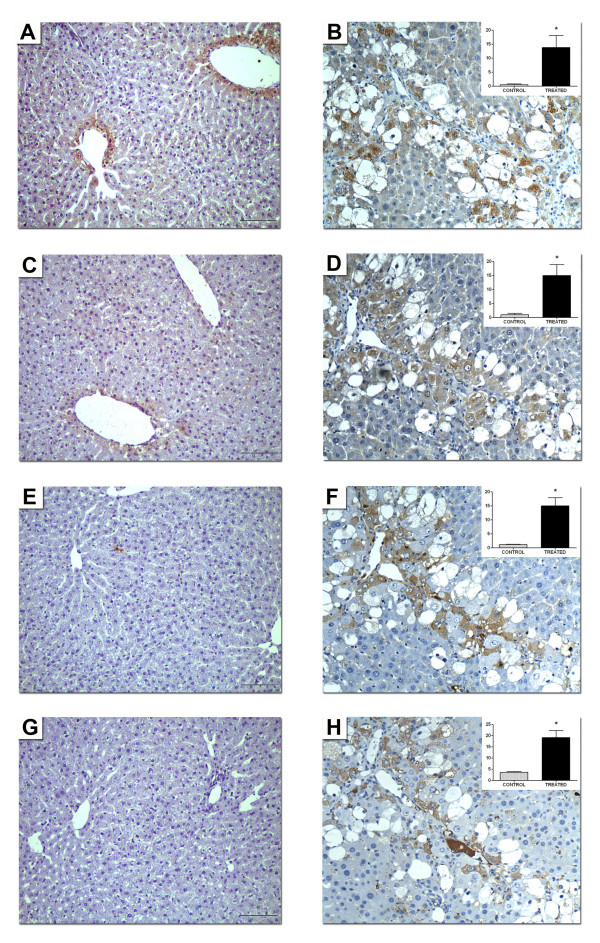
**Representative micrographs of protein expression (A and B) PON1; (C and D) MCP-1; (E and F) F4/80 and (G and H) 4-hydroxy-2-nonenal-protein adducts in liver tissue of control rats (A, C, E, G) and rats treated with CCl_4 _for up to 12 weeks (B, D, F, H)**. Original magnification: ×100. The inserts represent the mean percentages of positively-stained areas with respect to the total, in control and treated animals. * *P *< 0.001.

The hepatic immunoreactivities of PON1 and MCP-1 were significantly increased in CCl_4_-administered rats after 12 weeks of CCl_4 _exposure (Fig. 1). Of note is that the distribution was similar for both proteins. Hepatocytes were strongly stained when located in close proximity to inflammatory infiltrates (indicated by macrophage F4/80 antigen staining) and fibrosis septa. The expression of 4-hydroxy-2-nonenal protein adducts (an index of the degree of hepatic oxidative stress) was highest in these areas. The amounts of positively-stained hepatocytes for PON1 and MCP-1 were lower in the less-affected areas of the liver.

**Figure 2:**
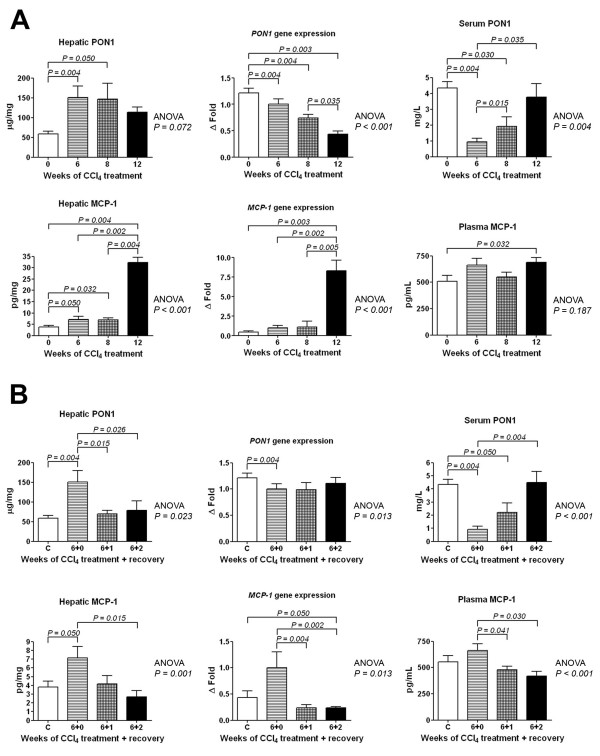
**Changes in MCP-1 and PON1 protein and gene expression with CCl_4 _administration (A) and with CCl_4 _withdrawal/recovery of liver function (B)**.

To extend these observations, we measured the hepatic and serum concentrations of PON1 and MCP-1 and their respective gene expressions. Hepatic PON1 concentration was significantly increased in CCl_4_-administered rats but, surprisingly, *PON1 *gene expression showed a significant decrease throughout the CCl_4 _exposure period. Serum PON1 concentration showed a significant decrease after 6 weeks of CCl_4 _exposure, followed by a subsequent increase to reach levels similar to those of control animals (Fig. 2A). Hepatic MCP-1 concentration and gene expression showed significant increases that were accompanied by moderate increases in plasma MCP-1 concentrations.

When we evaluated the recovery of liver histology and function following the cessation of CCl_4 _administration, we found that the hepatic and serum PON1 concentrations proceeded towards normal levels, and PON1 gene expression remained relatively unmodified (Fig. 2B). The recovery period was associated with a significant decrease in hepatic MCP-1 protein, MCP-1 gene expression, and plasma MCP-1 concentration.

Taking all the experimental data together (treatment + recovery), we observed significant direct relationships between hepatic PON1 concentrations, MCP-1 (*r *= 0.47; *P *= 0.005) and α-SMA staining (*r *= 0.55; *P = *0.001).

### Changes in hepatic PON1 expression are associated with nuclear transcription factors and decreased proteolysis

**Figure 3:**
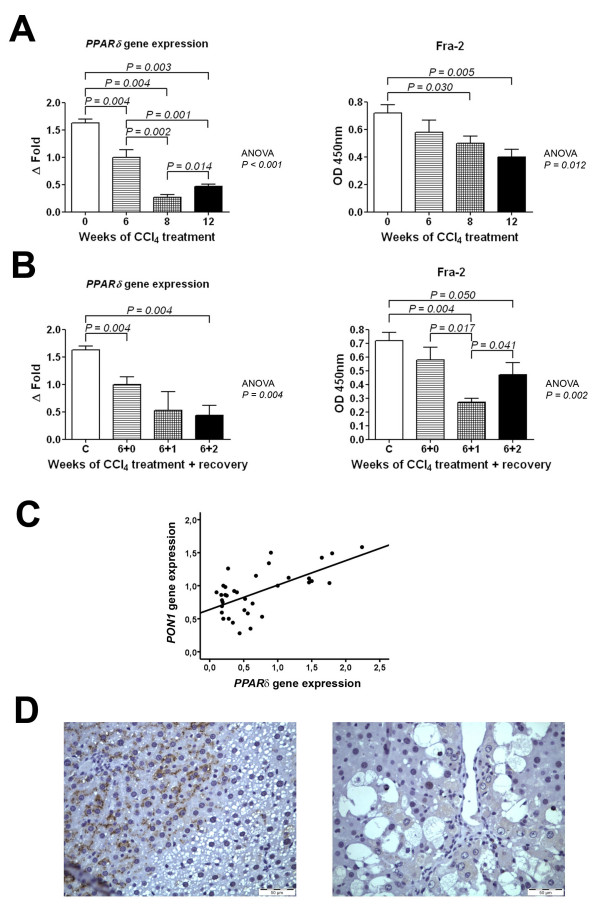
**Changes in *PPARδ *gene expression and Fra-2 activation over the period of CCl_4 _administration (A) and CCl_4 _withdrawal/recovery (B). **Panel (C) shows the relationship between *PPARδ *and *PON1 *gene expressions in all the animals studied (treatment and recovery). Panels (D) shows representative micrographs of PPARδ protein expression in liver tissue of control rats (left) or animals treated with CCl_4 _for 12 weeks (right). Original magnification: ×100.

To assess the molecular mechanisms of the apparently contradictory changes in the hepatic PON1 gene and its protein product expressions, we sought associations between nuclear transcription factors and the changes in PON1 gene expression. Hence, we analysed the levels of activated Sp-1 and those of several members of the AP-1 family, as well as the gene expressions of *PPARα*, *PPARδ *and *PPARγ *. We observed a strong decrease of *PPARδ *gene expression during the CCl_4 _exposure (Fig. 3A and 3B). Fra-2 changes followed a similar trend, albeit not so pronounced. We did not observe any significant change in Sp-1 or any of the other AP-1 proteins studied, nor in *PPARα *and *PPARγ *gene expressions (data not shown).

Taking all the experimental data together (treatment + recovery), we observed a significant direct relationship between *PPARδ *and *PON1 *(Fig. 3C) gene expression (*r *= 0.55; *P *< 0.001) but not between activated Fra-2 levels and *PON1 *(*r *= 0.27; *P = *0.121) or between Fra-2 and *PPARδ *(*r *= 0.26; *P = *0.127). PPARδ immunohistochemistry confirmed gene expression analysis, since it was clearly positive in control rats and faint in animals treated with CCl_4_for 12 weeks (Fig. 3D). These results suggest that PPARδ reduction may be involved in the molecular alterations underlying the inhibition of *PON1 *gene expression.

**Figure 4:**
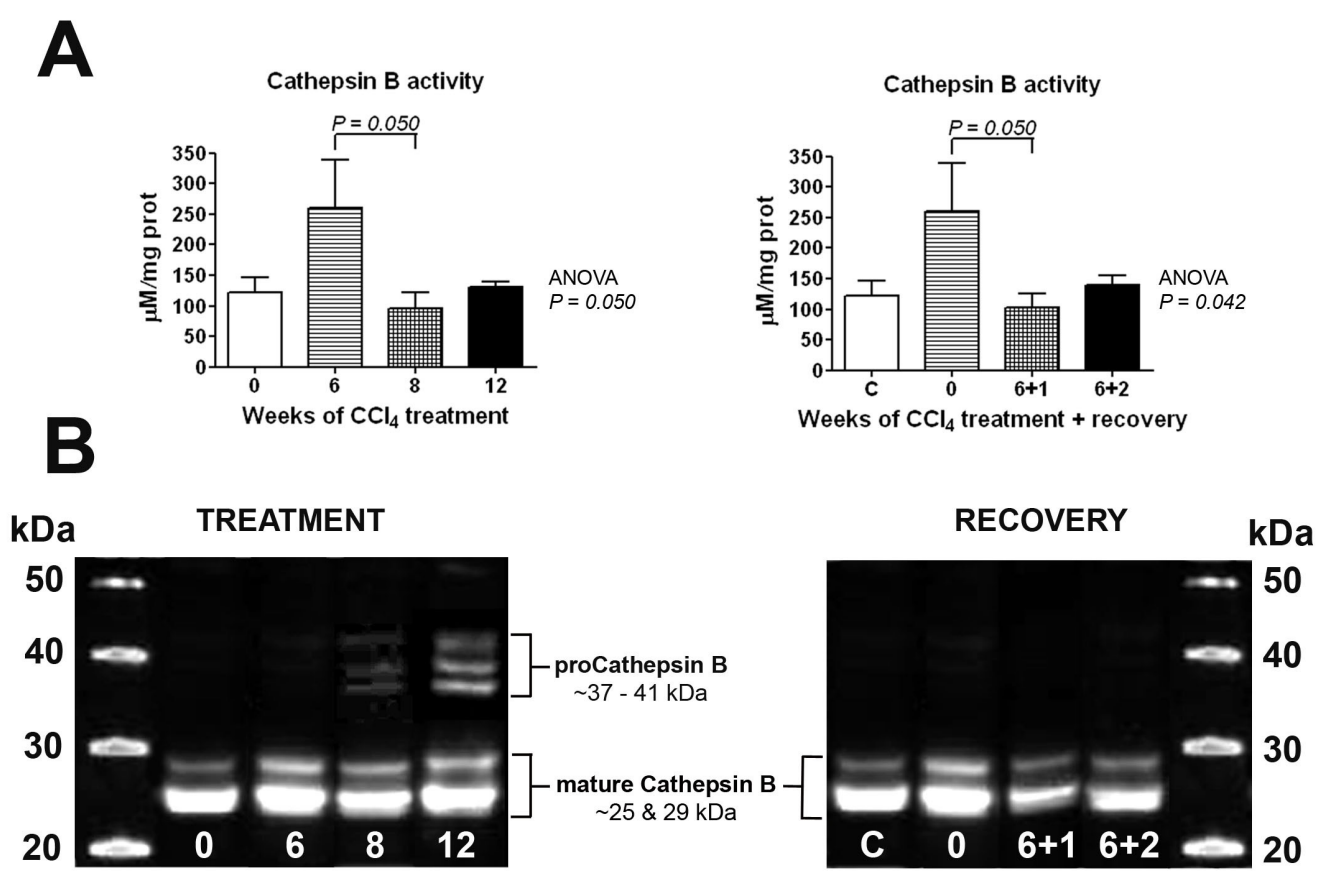
**Changes in (A) cathepsin B activity; (B) cathepsin B protein expression analysed by Western Blot over the period of CCl_4 _administration, and the subsequent withdrawal/recovery period**.

**Figure 5:**
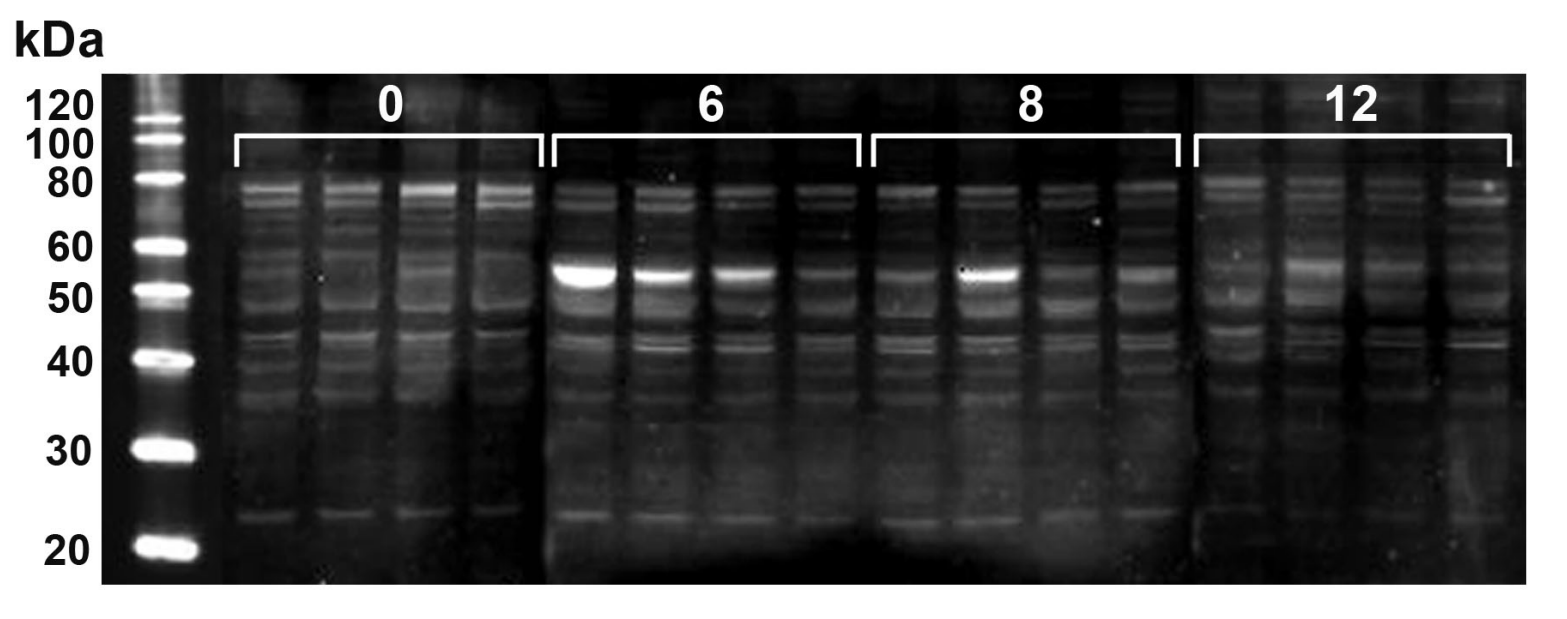
**Hepatic PON1 protein expression over the period of CCl_4 _administration**. Mature PON1 appears as a double band at ~40 kDa. Low-molecular weight immunoreactive fragments are clearly observed in control rats and in animals receiving CCl_4 _for 6 and 8 weeks, but their expression is decreased at week 12. In addition, the expression of a high-molecular weight (~55 kDa) unknown immunoreactive protein is enhanced in CCl_4_-administered animals at 6 and 8 weeks.

We then investigated the possibility that a decrease in PON1 protein degradation explains the increase in the intra-hepatic levels of this protein, despite the decreased gene expression. We estimated the liver proteolytic activity by measuring the protease cathepsin B, a reliable marker of hepatic cell proteolysis [26]. The hepatic cathepsin B activity was significantly increased after 6 weeks of CCl_4 _administration, followed by a decrease at weeks 8 and 12. This change was accompanied by the appearance, on Western Blot analysis, of bands corresponding to inactive procathepsin B (Fig. 4). We confirmed these results by performing Western Blot analyses of liver homogenates by using a specific anti-PON1 antibody. We observed that the presence of small immunoreactive fragments (MW lower than 35 kDa) was decreased after 12 weeks of CCl_4 _administration (Fig. 5).

### Changes in PON1 lactonase and esterase activities

**Figure 6:**
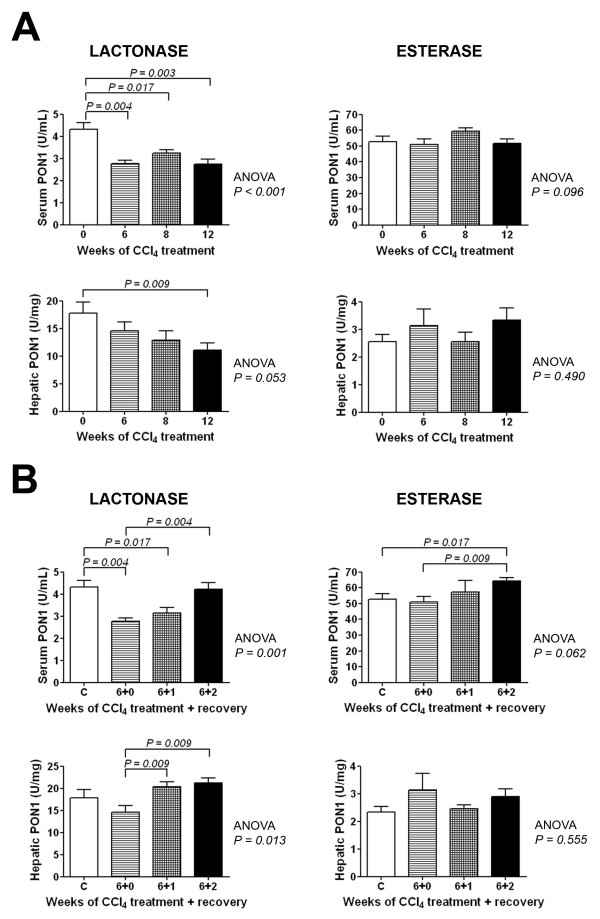
**Changes in the PON1 serum and liver lactonase and esterase activities during CCl_4 _administration (A) and recovery (B)**.

Serum and liver PON1 lactonase activities were consistently decreased throughout the exposure to CCl_4_, relative to control animals. We did not observe any significant change in PON1 esterase activity. The recovery of liver function was associated with a significant increase in serum PON1 lactonase activity, but PON1 esterase activity did not show any major alteration (Fig. 6). Hepatic PON1 lactonase activity was inversely related to hepatic MCP-1 concentration (*r *= -0.55; *P *= 0.001), and to the percentage of collagen in the liver biopsies (*r *= -0.37; *P *= 0.028), and to α-SMA staining (*r *= -0.61; *P *< 0.001). Serum PON1 lactonase activity was directly related to hepatic lactonase activity (*r *= 0.40; *P *= 0.017), and inversely to AST (*r *= -0.53; *P = *0.001), to the percentage of collagen (*r *= -0.46; *P *= 0.005), and to α-SMA staining (*r *= -0.69; *P *< 0.001). We did not find any significant relationship between PON1 esterase activity and any of these parameters.

## Discussion

The molecular mechanisms underlying the effects of CCl_4 _involve the production of the highly pro-oxidant trichloromethyl free radical (CCl_3 _*) and trichloromethylperoxy radical (CCl_3_OO*) which play key roles in the development of hepatic damage [27]. Reactive oxygen intermediates and aldehyde end-products of lipid peroxidation (such as 4-hydroxy-2-nonenal) act as mediators affecting signal transduction pathways as well as proliferation and functional responses of hepatic cells [28,29]. MCP-1 expression by the liver is up-regulated by 4-hydroxy-2-nonenal [30]. Our results show a significant increase in MCP-1 gene and protein expression in our experimental model, and which support previous studies in patients with chronic hepatitis or liver cirrhosis [31,32] in which MCP-1 expression correlated with monocyte infiltration in the liver. In these previous studies, the infiltration was observed in the portal tracts, the regenerating bile ducts, and in the septa surrounding the regenerating nodules, and was similar to the distribution of MCP-1 immunostaining observed in the present investigation. We also found that the histological and functional recovery following the CCl_4 _withdrawal was associated with a significant decrease in MCP-1 protein and gene expression.

MCP-1 is synthesised by a variety of hepatic cells including macrophages, endothelial cells, smooth muscle cells and hepatocytes [31]. The influence of MCP-1 on the histological alterations leading to liver impairment would appear to be crucial. This protein enhances hepatic stellate cell chemotaxis and contributes to the increase in collagen synthesis [33]. Further, MCP-1 knockout mice develop a lower degree of oxidative stress and accumulation of inflammatory cell infiltrate than their corresponding wild-type animals when receiving a single CCl_4 _dose [34]. Aldehyde products of lipid peroxidation and MCP-1 would appear to induce a synergistic effect on the fibrogenetic process leading from acute to chronic liver damage.

Several studies have investigated the role played by intracellular antioxidant defences in the protection against hepatic oxidative stress [35]. However, there is a paucity of information on the contribution of PON1 in this process. Recently, transgenic mice over-expressing human PON1 were shown to be partially protected against CCl_4_-induced liver injury [8]. PON1 attenuates MCP-1 production in cultured endothelial cells incubated with oxidised low-density lipoproteins [36]. This enzyme catalyses the hydrolysis of oxidised phospholipids and at least two of these phospholipids [1-palmytoyl-2-(5-oxovaleroyl)-*sn*-glycero-3-phosphorylcholine and 1-palmytoyl-2-glutaroyl-*sn*-glycero-3-phosphorylcholine] stimulate the production of MCP-1 [37]. In the present study, we observed a significant increase in the hepatic PON1 concentration following 6 and 8 weeks of CCl_4 _administration. During this period, MCP-1 hepatic concentration and gene expression were low. MCP-1 only increased around the 12^th ^week when PON1 concentration showed a trend towards a decrease. These data suggest that PON1 acts as a barrier against hepatic oxidative stress and, only when this barrier is overcome by protracted exposure to CCl_4_, does MCP-1 increase together with a concomitant development of a severe pro-inflammatory reaction. The histological examination of the liver biopsies in our present study suggests, as well, that the enhanced PON1 protein expression is related to the protection of hepatocytes from increased oxidative stress; PON1 staining being stronger in those cells surrounding the areas of 4-hydroxy-2-nonenal deposition and of macrophage infiltration. The recovery of liver function was associated with a significant decrease in hepatic PON1 protein expression.

The mechanisms underlying the regulation of PON1 and MCP-1 in this experimental model are complex. Of note was that despite observing an increased PON1 protein expression, *PON1 *gene expression was not enhanced but, rather, was decreased following CCl_4 _administration. These results may seem paradoxical, but we need to bear in mind that the intracellular accumulation of any protein is a result of a balance between synthesis, degradation, and secretion into the medium. Our results suggest that changes in PPARδ may be, at least in part, responsible for this alteration. PPARδ is the lesser known of the PPAR group of nuclear transcription factors. PPARδ increases apolipoprotein A-I and HDL synthesis by mechanisms that probably involve activation of the ATP-binding cassette transporter (ABCA1) gene [38]. We hypothesise that the decrease in *PPARδ *gene expression observed in the present study would be associated with a decreased HDL synthesis, which accords with the decreased serum cholesterol concentrations. This decrease would have, as a consequence, a decreased PON1 secretion to the extracellular medium. This would explain the decreased serum PON1 concentrations at weeks 6 and 8 of CCl_4 _administration (paralleling those of cholesterol) and the increased hepatic PON1 concentration. Moreover, PPARδ has been shown to up-regulate the expression of several antioxidant genes including superoxide dismutase, catalase and thioredoxin [39]. As such, a direct effect of PPARδ on the regulation of *PON1 *gene expression is a likely possibility.

**Figure 7:**
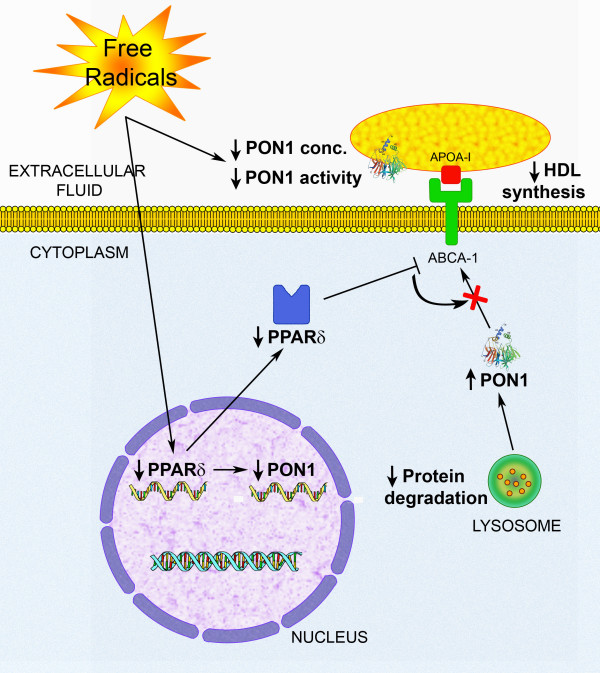
**A hypothetical biochemical pathway that could explain the PON1 alterations observed in the CCl_4_-administered rats**. Free radical-induced liver impairment would result in a decrease in *PPARδ *gene expression and, as a consequence, in *PON1 *gene expression. It would also induce an inhibition of ABCA1, a decrease in HDL synthesis and, therefore, a decrease in serum PON1 concentration. Serum lactonase activity would be decreased secondarily to these changes and, as well, due to a direct inhibition by free radicals. Intrahepatic PON1 levels would be increased as a consequence of a decreased protein degradation.

A precise explanation for PPARδ gene expression decrease in experimental liver disease is difficult. This transcription factor inhibits apoptosis in cultured cardiomyoblasts [40] and we have previously described hepatocyte apoptosis as being stimulated in experimental liver disease [41]. Probably, PPARδ levels decrease as a part of a pro-apoptotic mechanism tending to eliminate damaged hepatocytes during the process of liver damage. Within this context, hepatic PON1 levels continue to be elevated as a consequence of the combination of a decreased PON1 secretion into the HDL particles, essentially very early in the study, and to an inhibition of lysosomal protein degradation by the last weeks of the study. This enables the enzyme to continue playing its antioxidant and hepato-protective role. A scheme of the proposed mechanisms underlying the alterations in PON1expression in experimental liver disease is shown in Fig. 7.

A similar rationale to that of PPARδ may apply to Fra-2. This protein also stimulates ABCA1 gene expression [42], decreases cell apoptosis and stimulates proliferation [43]. However, our results show that Fra-2 decrease is lower than that of PPARδ, and does not correlate with PON1 gene expression. Probably, the contribution of this transcription factor to hepatic PON1 levels is, if any, modest or even indirect.

Hepatic and serum lactonase activity decreased during CCl_4 _administration and increased during the recovery period (CCl_4 _withdrawal). The finding of a reduced lactonase activity together with an enhanced hepatic PON1 protein expression is consistent with our previous studies in which we observed similar results in patients with chronic liver diseases [6]. The decreased lactonase activity is perhaps secondary to enzyme depletion following its reaction with products of lipid peroxidation. For example, there is evidence that PON1 is inactivated following the hydrolysis of lipid peroxides [44,45]. The changes in serum PON1 lactonase activity observed in our study were, in general, inversely correlated with the alterations in the concentrations of serum PON1 and MCP-1, and the degree of liver impairment. Conversely, the esterase activity, with paraoxon as the substrate, did not show any significant relationships with these parameters. Serum esterase activity is much lower in rats than in humans [5] and, in the light of the present results, we propose that it does not represent functional PON1 activity, but rather a more non-specific activity due, at least in part, to the action of other serum esterases, or to the esterase capability of serum albumin [46]. These results suggest that interpreting serum PON1 esterase activity would need to be conducted with caution in experimental studies in rodents.

## Conclusion

We suggest a hepato-protective role for PON1 against inflammation, fibrosis and liver disease mediated by MCP-1, and propose a hypothetical model to explain the hepatic PON1 alterations observed. Further studies with animals treated with artificial PON1-containing lipid constructs or with PPARδ agonists would be valuable in investigating measures to counteract the inflammatory and fibrogenetic processes in chronic liver impairment and, possibly, to provide new effective tools to treat this disease.

## Abbreviations

ABCA1: ATP-binding cassette transporter-1; AST: aspartate aminotransferase; ELISA: Enzyme-linked immunosorbent assay; MCP-1: monocyte chemoattractant protein-1; PCR: polymerase chain reaction; PON1: paraoxonase-1; PPAR: peroxisome proliferator-activated receptor; SMA: smooth muscle actin; TBBL: 5-thiobutyl butyrolactone.

## Competing interests

The authors declare that there have no competing interests.

## Authors' contributions

JM, study design, performed experimental studies, statistical analysis, drafted the manuscript; JC, study design, edited the manuscript; NF, study design, performed experimental studies; RB, immunohistochemistry, nuclear transcription factor analyses; AR, rat breeding, immunohistochemistry; BM & MM, generation of anti-PON1 antibodies, contribution to study design, edited the manuscript; JJ, edited the manuscript. All authors read and approved the final manuscript.

## Pre-publication history

The pre-publication history for this paper can be accessed here:

http://www.biomedcentral.com/1471-230X/9/3/prepub
